# The effectiveness of MyBFF@school intervention program in reducing emotional and behavioral problems in overweight and obese secondary schoolchildren in Malaysia: a cluster randomized controlled trial

**DOI:** 10.1186/s12889-025-23545-y

**Published:** 2025-07-28

**Authors:** Zahari Ishak, Low Suet Fin, Wan Abdul Hakim Wan Ibrahim, Abqariyah Yahya, Fuziah Md. Zain, Rusidah Selamat, Muhammad Yazid Jalaludin, Abdul Halim Mokhtar

**Affiliations:** 1 Wellbeing Research Center, Faculty of Social Sciences and Liberal Arts, UCSI University, Kuala Lumpur, 56000 Malaysia; 2 School of Education, Faculty of Liberal Art, Humanities and Culture, Perdana University, Kuala Lumpur, 50490 Malaysia; 3 Department of Social and Preventive Medicine, Faculty of Medicine, Universiti Malaya, Kuala Lumpur, Wilayah Persekutuan Kuala Lumpur 50603 Malaysia; 4 Department of Pediatrics, Putrajaya Hospital, Ministry of Health Malaysia, Jalan P9, Pusat Pentadbiran Kerajaan Persekutuan Presint 7, Putrajaya, Wilayah Persekutuan Putrajaya 62250 Malaysia; 5 Nutrition Division, Ministry of Health Malaysia, Level 1, Block E3, Complex E, Federal Government Administrative Centre, Putrajaya, Wilayah Persekutuan Putrajaya 62590 Malaysia; 6 Department of Pediatrics, Faculty of Medicine, Universiti Malaya, Kuala Lumpur, Wilayah Persekutuan Kuala Lumpur 50603, Malaysia; 7 Department of Sports Medicine, Faculty of Medicine, Universiti Malaya, Kuala Lumpur, Wilayah Persekutuan Kuala Lumpur 50603 Malaysia; 8 Department of Educational Psychology and Counselling, Faculty of Education, Universiti Malaya, Kuala Lumpur, Wilayah Persekutuan Kuala Lumpur 50603 Malaysia

**Keywords:** Anxiety, Depressed, Obese, Somatic Complaints, Thought Problems, Withdrawn

## Abstract

**Background:**

Obesity may have negative impacts on the physical and psychosocial aspects of children. This study aimed to assess the effectiveness of the MyBFF@school intervention program on emotional and behavioral problems among overweight and obese secondary school children in Malaysia.

**Methods:**

Children were assessed using the Youth Self-Report Questionnaire to measure their emotional and behavioral problems. Comparisons between the intervention group and control group after a six month intervention period were analyzed using linear mixed-effect model.

**Results:**

Altogether, 768 children were recruited, 447 in the intervention and 321 in the control group. There were slight reduction in majority of YSR scales within the intervention group but there was no statistically significance different comparing intervention and control groups at baseline and at 6-months follow-up.

**Conclusions:**

The findings demonstrated a feasibility of implementing a multifaceted intervention program in school children with obesity in Malaysia. More holistic and perhaps longer intervention period needed to improve the outcomes significantly among children with obesity. The findings demonstrate the importance of psychology components in intervention programs combating obesity among overweight and obese secondary school-aged children.

**Trial registration:**

Clinical trial number: NCT04155255, November 7, 2019 (Retrospective registered). National Medical Research Register: NMRR-13–439-16,563. Registered July 23, 2013. The Medical Research and Ethics Committee (MREC), Ministry of Health Malaysia and Educational Planning and Research Division (EPRD), Ministry of Education Malaysia approved the intervention program. It was funded by the Ministry of Health Malaysia.

## Background

Childhood obesity has become a global epidemic with increasing prevalence since 1990, particularly in developed countries and in major cities of developing countries [1]. It is a matter of great concern worldwide for its effects on the physical and mental health of children. Overweight children suffer from metabolic syndrome, asthma, dental problems, and psychological problems [2]. A systematic review among Australian children and adolescents found evidence that obese children and adolescents had more psychological problems compared to their normal weight counterparts [3]. Another study found an association between overweight and obese children and psychological problems, such as low self-esteem, depression, poor health-related quality of life, and emotional and behavioral disorders [4].

These problems require mitigation since self-esteem is inversely related to depression and anxiety, whereas depression is interrelated with certain somatic complaints [5, 6]. Moreover, children with low self-esteem may develop social problems in adulthood [7]. Mass media exerts further pressure on these children by giving an ideal image of thin bodies, leading overweight and obese individuals to develop a negative body image and disordered eating patterns [8]. Moreover, these children often faced weight-based stigma and bullying, leading to social isolation [9]. Poor relationship status with their peers could also increase the prevalence of children exhibiting depression, anti-social behavior, and social anxiety [10, 11].

**Fig. 1:**
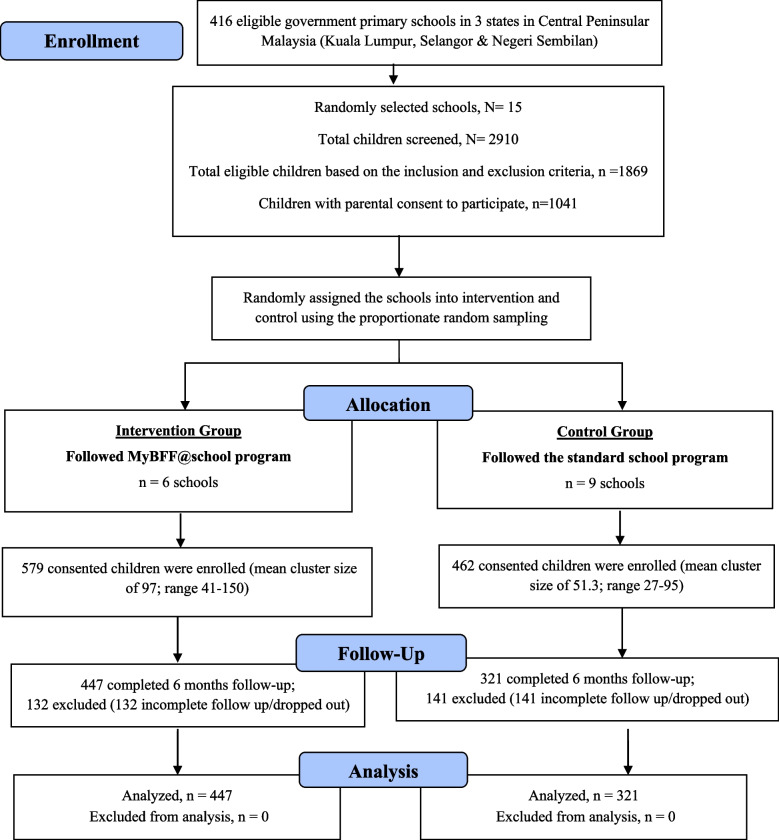
CONSORT diagram for psychology component in MyBFF@school

The emotional and behavioral problems faced by these children demand stringent measures. Childhood obesity intervention programs have been implemented across the world using many methods with varying degree of success. A meta-analysis found that school-based interventions are effective in combating childhood obesity. Previous interventions have usually targeted increased physical activity, dietary behavior, or a combination of both [12]. However, there is a paucity of information on childhood obesity interventions that incorporate psychological approaches to address the emotional and behavioral problems leading to the birth of this MyBFF@school program. The main objective of this study was to investigate the effectiveness of the MyBFF@school obesity intervention program in ameliorating emotional and behavioral problems among overweight and obese secondary school children (Fig.1).

## Methods

The design and methodology of this study are described at length by Mokhtar et al. [13]. The demography of participants is shown in Table 1. Researchers collected data from participating children at study baseline and at the end of the 6-month intervention. Data were collected through a self-reporting questionnaire as well as via anthropometric measurement. We received written informed consent from the parent or guardian and assent from the children participating in the study. From 1041 (35.8%) children, only 768 (73.8%) children completed the questionnaire for the psychology component, where 447 children were in the intervention group and the remaining 321 children were in the control group. About 26% of the children have dropped out from the program due to scheduling conflicts and lack of interest.

**Table 1:** Demography of participants

Characteristic of respondents	Intervention *N* = 447	Control *N* = 321	Chi-square	*p*- value
Gender, *n* (%)				
Boys	172 (38.5)	129 (40.2)	0.229	0.632
Girls	275 (61.5)	192 (59.8)		
Location, *n* (%)				
Urban	264 (59.1)	246 (76.6)	25.869	< 0.001*
Rural	183 (40.9)	75 (23.4)		
Age groups, *n* (%)				
13 years	140 (31.3)	92 (28.7)	4.397	0.355
14 years	110 (24.6)	91 (28.3)		
15 years	2 (0.4)	1 (0.3)		
16 years	191 (42.7)	137 (42.7)		
17 years	4 (0.9)	0		
Ethnicity, *n* (%)				
Malay	370 (82.8)	229 (71.3)	22.376	< 0.001**
Chinese	27 (6.0)	31 (9.7)		
Indian	40 (8.9)	59 (18.4)		
Others	10 (2.2)	2 (0.6)		
Body weight groups, *n* (%)				
Overweight	195 (43.6)	170 (53.0)	6.529	0.011*
Obese	252 (56.4)	151 (47.0)		

*p < 0.05

**p < 0.01

The psychological module comprised of five aspects (self-esteem, friendship, assertive, positive thinking for healthy life, and stress management). This module had been adopted and adapted by a team of researchers based on literature review and developmental theories [14]. The module aimed to build the children’s self-confidence and resilience in facing challenges during the intervention and to minimize dropout rate. Since overweight and obese children often face stigma because of their body weight, [9] the module is crucial to boost their will power in order to sustain and complete the program.

## Instrument

Data on symptoms of emotional and behavioral problems are gathered using the Youth Self-Report (YSR) questionnaire. The YSR questionnaire was derived from the Child Behavior Checklist first developed in 1991 by Thomas M. Achenbach [15]. Scores were obtained from the examined items using eight narrow-band scales (withdrawn, somatic complaints, anxious/depressed, social problems, thought problems, attention problems, rule-breaking behavior, and aggressive behavior) and three broad-band scales (internal problems, external problems, and total behavior) [15]. The questionnaire contains 112 items, for which the children were asked to rate each as zero (not true), one (somewhat/sometimes true), or two (often/really true) [16].

The content validity of the questionnaire was proven using previous findings and supported by four decades of research, consultation, feedback, and revision. The validity of the questionnaire was supported in four ways, namely, proof of significant association with the Diagnostic and Statistical Manual of Mental Disorders criteria and other questionnaires of similar scale, the Achenbach System of Empirically Based Assessment (ASEBA) syndrome cross-cultural replication, genetic and biochemical evidence, and prediction of consequences over long periods of time [17].

The Cronbach’s alpha value of the questionnaire during a pilot study was determined to be 0.70. In this study, the overall Cronbach’s alpha value of 0.95 proves the reliability of the instrument. The reliability coefficients for the YSR subscales were as follows: Anxious/Depressed (α = 0.85), Withdrawn/Depressed (α = 0.79), Somatic Complaints (α = 0.74), Social Problems (α = 0.78), Thought Problems (α = 0.81), Attention Problems (α = 0.84), Rule-breaking Behavior (α = 0.76), and Aggressive Behavior (α = 0.88). Levene’s test showed that the variances for attention problems (F(3,737) = 5.396, *p* = 0.001) and rule-breaking behavior (F(3,738) = 3.088, *p* = 0.027) were not equal. Assumptions of equality of variances were met for all other symptoms.

## Statistical analysis

Statistical distribution for all outcomes were determined. Kolmogorov–Smirnov test (*p*-value > 0.05) and skewness index (acceptable limits of ± 2) were determined and data that follows the assumption is considered as normally distributed. Continuous variables are presented as mean values. Chi-square tests of homogeneity were used to determine the distribution of the children across different variables. The effectiveness of the program was measured using linear mixed-effect model adjusting for baseline values, treatment group, gender and school location (urban or rural). While school is considered as clustering effect. The IBM Statistical Package for the Social Sciences (SPSS) version 24 (SPSS Inc., Chicago, IL, USA) and Stata version 14 (StataCorp. 2015. Stata Statistical Software: Release 14. College Station, TX: StataCorp LP) were used to estimate all models. All statistical tests were conducted at the 5% significance level.

## Results

The mean age (SD) of the children participating in this study was 14.57 (1.32) years. A chi-square test showed that there were statistically significant differences in school location (X^2^(1) = 25.869, *p* < 0.001) and ethnicity (X^2^(3) = 22.376, *p* < 0.001), i.e., there were fewer children from rural schools in the control group. Majority of the children in this study were of Malay race, and most were in the overweight and obese categories. On the other hand, the chi-square test showed no significant differences in gender, age groups, and body weights of the children. The test showed that the children had similar characteristics across these three socio-demographic characteristics.

There were no statistically significant differences between intervention and control groups in any of the eight narrow-band scales comparing baseline and six months. We also observed that the magnitude of the differences between control and intervention groups were very small and all 95% confidence intervals crosses null value (Table 2).

**Table 2:** Adjusted differences for all YSR scales between control and intervention groups at baseline and six months follow-up

	Intervention, *N* = 447		Control, *N* = 321		Mixed Linear Model		
	Mean (SD) baseline	Mean (SD) month-6	Mean (SD) baseline	Mean (SD) month-6	Mean difference (SE) adjusted	95% CI	ICC
Anxious/depressed	7.840 (4.338)	7.408 (4.212)	7.378 (4.229)	7.481 (4.457)	−0.205 (0.414)	(−1.018, 0.606)	0.014
Withdrawn/depressed	5.360 (2.821)	4.914 (2.810)	5.001 (2.821)	5.213 (2.888)	−0.325 (0.298)	(−0.911, 0.259)	0.018
Somatic Complaints	4.995 (3.381)	4.538 (3.116)	4.712 (3.229)	4.490 (3.346)	−0.150 (0.292)	(−0.723, 0.422)	0.007
Social Problems	6.449 (3.399)	6.223 (3.357)	6.142 (3.484)	6.366 (3.654)	−0.166 (0.342)	(−0.837, 0.503)	0.014
Thought Problems	5.884 (3.807)	5.901 (3.832)	5.793 (3.804)	6.030 (4.190)	−0.261 (0.367)	(−0.982, 0.458)	0.010
Attention Problems	6.764 (3.072)	6.771 (3.079)	6.298 (3.058)	6.648 (3.173)	0.027 (0.362)	(−0.683, 0.737)	0.029
Rule-breaking Behavior	4.606 (3.586)	5.015 (3.624)	4.309 (3.835)	4.816 (3.768)	0.060 (0.334)	(−0.594, 0.715)	0.009
Aggressive Behavior	9.241 (5.071)	9.417 (5.239)	8.545 (5.288)	9.374 (5.535)	−0.069 (0.526)	(−1.102, 0.962)	0.015

Model adjusted for baseline value, gender, school and location

## Discussion

The general aim of the MyBFF@school study was to introduce a multifaceted school-based intervention for both primary and secondary school children with obesity problem in the central region of Peninsular Malaysia. This study was supported by Ministry of Education, Ministry of Health as well as the Malaysian Industry-Government Group for High Technology (MIGHT) under the Prime Minister Office. The entire intervention program was developed by own researchers in this study and was delivered by well trained personnel in each school. The primary aim of this report was to reduce all scales within the Youth Self-Report (YSR) questionnaire. This aim however was not achieved and mean difference between groups for all scales were not statistically significant at the end of the follow-up period.

Studies have shown that support from friends is vital in facing difficulties and challenges [18]. A previous study found that high conflict or low support in friendship increases depression in children regardless of gender [10]. Another study also revealed that relationship with peers is strongly linked to social anxiety [11]. The friendship aspect aimed to improve such relationships through mutual understanding. Friendships could be deepened, and new friends could be made through the activity sessions, which could reduce feelings of anxiety and depression. The children were also taught how to manage stress through problem sharing or physical activities, since stress can contribute to depressive moods and avoidance, both of which can lead to unhealthy eating habits [19].

Improving self-esteem through the module is beneficial for participating in reducing anxiety, depression, and feelings of withdrawal. It could assist participants in identifying their strengths and weaknesses in order to make them value their self-worth and increase their self-confidence. This is important for their emotional health as self-esteem, is inversely related to having anxiety or depression [5, 20]. Lower self-esteem could also lead children to have social problems in adulthood [7]. Thus, increasing self-esteem in youth is imperative for future psychological development by preventing children from becoming withdrawn at an early age. Medically unexplained somatic symptoms among children showed significant improvements at the end of the program. A previous study revealed that, even though causal relationships could not be established between somatic complaints and anxiety and depression, there is strong evidence for interrelationships between them [21]. Since the program has potential benefits on both anxiety and depression, children that join the program also reported less somatic complaints at the end of the intervention because of a lower level of anxiety and depressions.

Tremblay and Lariviere found that peer pressure is among the main predictors of disordered eating behavior [22]. Weight-based teasing can cause negative body image perception in children and inadvertently result in disordered eating behavior. On the other hand, body image pressure to be thin from mass media is identified to be a predictor of both negative body image and disordered eating [8]. Disordered eating might increase attention problems among children as noted by Kaisari et al. in a study showing that there is evidence for a positive association between ADHD and disordered eating, particularly overeating disorder [23]. The psychology module of the MyBFF@school program equipped participants with skills to cope with stress and improve their self-esteem. Such skills could help them deal with the urge to practice unhealthy eating habits. The nutrition module also increases awareness on the importance of healthy eating habits. Problems related to a reduction in attention among children could be mitigated by the acquisition of the aforementioned skills and awareness from this program.

The reduction of social problems among children could be an indication of the success of the aspects of friendship and assertive behavior in the psychology module. The friendship aspect of the module teaches children must-have skills in maintaining good relationships. On the other hand, the assertive behavior module may help them deal with negative situations in relationships with their peers. These skills could be crucial in empowering children to develop better relationships at school which can, in turn, reduce social problems. Moreover, thought-related problems could reduce as a result of improved relationships between children. Such improvements can prevent social isolation associated with thought problems such as self-harm [24].

Being a victim of bullying is not unusual for overweight and obese children [25]. However, studies have shown that severely obese male children tend to be both the victims and the perpetrators of bullying, [26] the latter of which may reflect previous experience as victims. Boja suggested inducing assertive behavior to prevent bullying [27]. However, it appears that the assertive aspect of the psychology module does not have a significant effect in improving behavioral problems among children. This may be because children with obesity that perpetrate bullying were not taken into account in the assertive aspect, as it was designed specifically to address victims.

The MyBFF@school is a large childhood obesity intervention conducted within a multiracial and socioeconomically diverse population. The intervention was designed as multifaceted program to cover a broad area of children livelihood based on a vast and promising evidence found from systematic review as well as a broad range of behavioral change studies. To our knowledge this study is the first in Malaysia focusing on children with obesity problems and conducted as cluster RCT. The analysis took the clustering effect and we believed that the findings were reliable. There was a slight reduction in the score of several domains (anxious/depressed, withdrawn/depressed, somatic complaints and social problems within the intervention group comparing baseline and at six months. While within the control group, majority of scales were observed to increase comparing baseline and at six months.

Nevertheless, there were also some limitations in this study. The exclusion of children with normal BMI-for-age from this program eliminated the possibility of educating them to the detrimental effects of stigmatization of peers suffering from obesity and the importance of social support for these peers [9, 28]. Considering the Whole School, Whole Community, Whole Child model in improving the health of children by involving all children in the program [29], the likelihood of reducing emotional and behavioral problems among children could be improved by following the approach in that model.

Another limitation of this study is the choice of an assessment tool to infer emotional and behavioral problems in children. A further limitation is the varying degrees in the implementation of the intervention due to different pre-existing facilities in the schools (such as a field for Small Sided Games (SSG) as well as differences, albeit minor, in how each of the trained personnel carried out the intervention. Although we reported the size of treatment effect as small based on adjusted mean difference of interpretation, the fluidity of psychological research requires a non-standardized approach to reasonably match our research question [30]. The MyBFF@school intervention program could be further improved by taking social environments into account. Studies have found that weight-based bullying still persists up to two years after obesity intervention programs were implemented in schools [31] and may result in emotional and behavioral problems that either remain unchanged or worsen.

## Conclusions

The MyBFF@school program showed positive changes in the majority of YSR scales within the intervention group but no statistically significant difference between groups after six months. Reviewing the module and redesigning activities focusing on additional symptoms could improve all scales. The findings also demonstrated that it is essential to include a psychology component in school-based programs combating obesity among overweight and obese children in order to reduce negative psychological effects.

## Data Availability

All relevant data are within the paper.
